# Supplementary material for: COVID-19 and Diagnostic Testing for SARS-CoV-2 by RT-qPCR—Facts and Fallacies
Source: Int J Mol Sci. 2021 Feb 28;22(5):2459. doi: 10.3390/ijms22052459 (PMC7957603; doi:10.3390/ijms22052459)
Supplement: Supplementary file 1 [file ijms-22-02459-s001.pdf]

Supplementary Information

Materials and Methods

RNA

Some of the same RNA samples described previously {Bustin et al., 2020, #78972} were used to demonstrate Cq variability.

Primers and probes

The primers and probes are shown in the table below, with their detailed properties described earlier {Bustin et al., 2020, #78972}.

Target	Target	Amplicon	Oligonucleotides (5'-3')	Final conc
SARS-CoV-2	Nsp10	85 bp	F: GGATCAAGAATCCTTTGGTGG	1 µM
			R: GTCACAAAATCCTTTAGGATTTGGA	1µM
			Pr: FAM-CATCGTGTTGTCTGTACTGCCGTTGCC	0.4µM
	N-gene	88 bp	F: GCTGCTAGACAGATTGAAC	1 µM
			R: AGCAGATTTCTTAGTGACAGTTTG	1µM
			Pr: FAM-TCTGGTAAAGGCCAACAACAACAAGG	0.4µM
	Nsp12	96 bp	F: CATCCCTACTATAACTCAAATGAA	1 µM
			R: GTCATAGTACTACAGATAGAGACAC	1µM
			Pr: FAM-TGCAAAGAATAGAGCTCGCACCGT	0.4µM

RT-qPCR reactions

All reactions were carried out in duplicate using 1µL of RNA in 5µL RT-qPCR reactions using either individual Nsp12 or N-gene (Figure 1A) or the triplex (Nsp10, 12 and N-gene) CoV2-ID assays (Figures 1B and 2). Following a 5-minute RT reaction at 50ºC and a 1-minute activation at 95ºC, PCR reactions were carried out for 40 cycles of 5 second denaturation 10 seconds polymerisation. The three mastermixes used had been previously validated to work well under these conditions.

Results

Figure S1

This shows the variability of Cqs associated with the use of different kits and instruments. A comparison of seven RT-qPCR master mixes used to detect two SARS-CoV2 targets results in Cqs ranges for Nsp12 and N of 3.25 and 4.43, respectively, which translate into apparent 9- and 22-fold differences in viral load (Figure S1A). Since both assays were extensively optimised, there was comparatively little assay-dependent variation, with a median ΔCq of 0.86 (range 0.09-1.8). The different Cqs associated with the choice of qPCR instrument are illustrated in Figure S1B. Cqs were recorded for two samples, each amplified by three different RT-qPCR master mixes and run on three separate instruments. It is evident that the instruments return diverse Cq values, with the maximum ΔCq being 5.05 (sample 2 MM1), which translates into a 33-fold difference. Again, it is notable that different master mixes perform differently, with maximum ΔCqs for the two samples across the three instruments of 7.28 and 9.17, the equivalent of 150 and nearly 600-fold differences in apparent viral load.

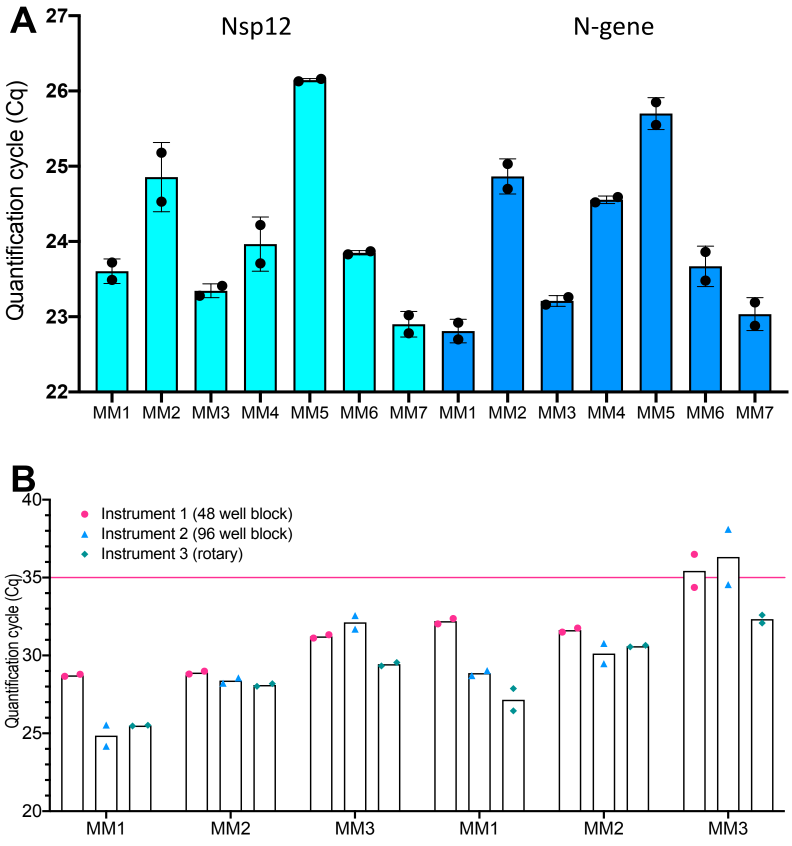

Figure S1. Master mix and instrument-associated Cq variability. **A.** Cqs recorded for Nsp-12 (light blue) and the N-gene (dark blue) using seven master mixes (MM1 to MM7) and the same RNA sample on a PCRMax Eco qPCR instrument. Default instrument threshold and baseline settings were used. Individual Cqs and means ± SD are shown. **B.** Two samples were amplified using three master mixes on 48 well (pink) and 96 well (blue) thermal block and 48 reaction rotary magnetic induction (green) qPCR instruments. The horizontal pink line indicates the position of Cq 35.

Figure S2

Differences in Cq due to threshold or instrument variability are shown in Figure S2. Operator adjustment of the default threshold results in a median decrease of 2 Cqs (range 0.65-4.84), which suggests an equivalent of a 1.5-30-fold difference in apparent viral load (Figure S2A). The data also demonstrate how operator intervention can lower Cqs below the proposed Cq of 35, as in this case, or result in more samples being above that Cq, had the threshold been lowered. Finally, the range of Cqs generated by changing thresholds is illustrated in Figure S2B, which shows a box and whiskers plot delineating the Cq range achieved by arbitrarily moving thresholds. The results are derived from three master mixes and two RNA samples, with the median Cq being 3.3 (range 2.48-3.89), a 6-15-fold difference in apparent viral load. The inclusion of standards and controls would allow disparate Cqs to be normalised to those standards and controls, so facilitating a comparison between results obtained in different laboratories using different reagents and instruments. This would then allow a correlation to be made between the relative Cqs recorded and the clinical significance of that result.

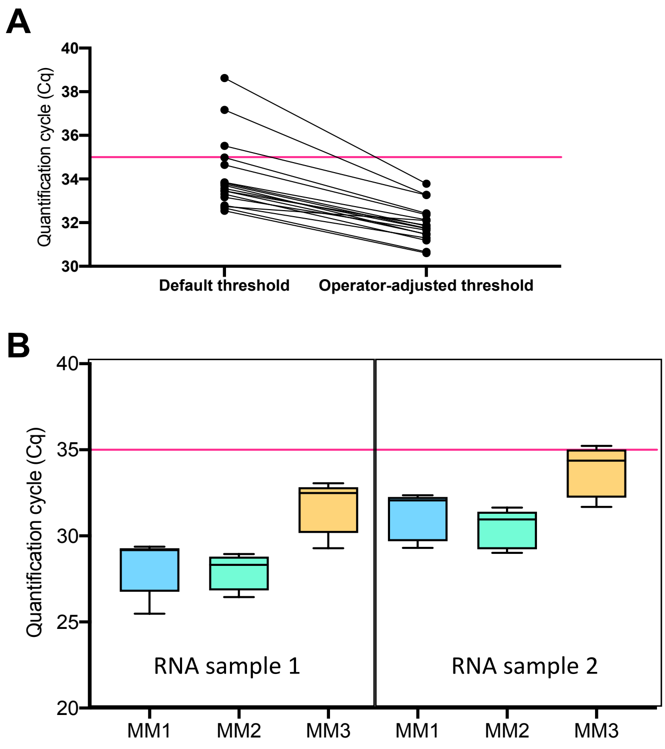

Figure S2. Threshold-associated Cq variability. Reaction conditions were the same as described in Figure 1 using CoV2-ID. **A.** 18 RNA samples were amplified using the same master mix, with default Cqs recorded by the instrument compared to those obtained when the threshold was changed by the operator. The horizontal pink line indicates the position of Cq 35 **B.** Two RNA samples (1 and 2) were amplified using three master mixes, shown in blue, green and orange, and Cqs obtained using default, minimum and maximum thresholds were plotted, with the whiskers showing the minimum and maximum Cqs. The horizontal pink line indicates the position of Cq 35.
